# Responsibility to defend Earth as a core principle of the planetary defense security regime

**DOI:** 10.1038/s41467-024-51517-0

**Published:** 2024-08-19

**Authors:** Nikola Schmidt

**Affiliations:** Institute of International Relations, Nerudova 3, Prague 1, Czech Republic

**Keywords:** Social sciences, Politics, Planetary science

## Abstract

This manuscript proposes the Responsibility to Defend Earth (R2DE) as a core principle for planetary defense, aiming to foster international cooperation and policy changes. Despite technical advancements and creation of bodies like IAWN and SMPAG, it highlights the need for consensus on action and means.

## Teaser

The planetary defense community should consider the development of a Responsibility to Defend Earth (R2DE) as a foundational normative principle for a future planetary defense security regime. This requires thorough deliberation and consensus-building to definitively answer the question: “What is the value we secure?”

In recent decades, the planetary defense community has performed a considerable job in creating procedural components of a security regime by building a rational foundation for understanding the asteroid impact risk and demonstrating our capability to respond to it. While this new knowledge rearticulates states’ responsibility, countries have not reached an internalized consensus over the necessity to act and the appropriate means of action. To broaden the dialogue from just the technical aspects of our response to include the non-contentious policy options, we must analyze, comprehend, and reach a consensus on the very foundation of the problem – what we intend to secure – and stipulate it in a shared norm as a foundational element of the future security regime.

Planetary defense inherently poses a challenge on a planetary scale that stands to benefit from broad international cooperation. To garner cooperation in this field, the countries need to reconcile their divergent interests and ensure to avoid misunderstandings that may arise from dual-use technologies or ambiguous intentions. At the same time, they should encourage cooperation even in the absence of immediate threats and reach a consensus on the notion that cooperating on planetary defense is sensible because it ensures security, stimulates economic growth, and fosters scientific and industrial collaborations. These objectives can be realized through the establishment of a planetary defense security regime that incorporates procedural, cognitive, and normative components [Box 1].

Regimes are intentionally created partial international orders to remove an issue area from the domain of self-help^1^ by establishing “a set of principles, norms, rules, and decision-making procedures around which actors’ expectations converge in a given area of international relations”^2,3^. Regimes could emerge from informally interconnected agents such as epistemic communities^4^ (transnational expert networks) and translate into formal regimes based on rules and procedures. However, here I use various definitions and conceptualizations of security regimes to argue that we need rules and procedures, achieve a mutual understanding, and reach a consensus represented by the three components mentioned above and described in Box 1.

I intend to look at the current planetary defense governance and show how the procedural components (procedures coordinating actors to achieve certain tasks–following observations of a found asteroid to successfully track it and locate its impact) of the emerging planetary defense regime are being developed by experts. I discuss how political authorities slowly try to find consensus on cognitive components (e.g., threat comprehension, the reasoning of necessity, chosen appropriate means, relation to national security, economic, and research and development priorities), while the normative components (e.g., sense of necessity, logic of appropriateness, arguments of responsibility and values to be secured) are neglected. Such normative components can be developed by the planetary defense epistemic community to stimulate better cognitive reasoning by political authorities.

## Box 1 Components of the planetary defense regime

*Procedural components* are instrumental in circumventing issues, reduce transaction costs in diplomacy and resolve game theoretical problems. They could also play a constitutive role in establishing states’ responsibilities through the production of knowledge. When established in advance, these procedures ensure the predictability of political actors making predictable decisions in planetary defense mission options. Consequently, procedures limit the political maneuverability of these actors, which might be advantageous for international cooperation, although not necessarily for ambitious political individuals. “Procedures have the potential to prevent internationally undesirable actions, such as the use of nuclear explosive devices, while facilitating timely decisions about less effective but longer-term solutions, like multiple kinetic impactors. Nonetheless, procedures could assist in drafting elements for the UN Security Council’s decision to proceed with the deflection mission.

*Cognitive components* of regimes help create a shared foundation for the overall understanding of the endeavor. These elements become particularly crucial when humanity isn’t being directly threatened by a cataclysmic asteroid, or when the threat is not confirmed. During this time, many individuals may dismiss the potential danger as unlikely due to statistical probability. However, these components may also facilitate achieving consensus within the Security Council at times when urgent resolution is necessary. It could further preemptively label opposition as irresponsible, as the functionality of these cognitive aspects rely on suitable actions derived from the regime’s normative components.

*Normative components* help to gain interest in the issue – spark cognitive reasoning – and stimulate international support for the emergence of the regime. Most importantly, the norms in emerging regimes assist in reaching a consensus on the moral responsibility to address pressing issues. Herein lies the opportunity for the planetary defense community to make the case that, considering the knowledge acquired, they expect authorities to uphold their responsibilities as defined under the social contract. In this context, norms play a pivotal role in shielding what humanity holds dear.

## The current state of planetary defense activities

To achieve widespread agreement, extensive social learning is essential for fostering a mutual understanding. Questions such as “What influence does planetary defense technology exert on national power due to its potential dual-use applications?”, “How can domestic businesses contribute?”, or “What moral obligations would states assume?” are essential. The present configuration of planetary defense governance lacks sufficient mechanisms to preclude an undesirable unilateral mission, or to dissuade a powerful state from unilaterally and legally acting. This situation isn’t optimal for guaranteeing Earth’s defense while maintaining international security and global political stability.

On the technical side, the planetary defense scientific community has performed considerable work in building a rational foundation for understanding asteroid impact risks, and in demonstrating via Double Asteroid Redirection Test (DART) mission our technical capability for their deflection. The same community has been interacting with world political figures and international institutions to establish a planetary defense governance system to ensure a smooth flow of decisions in case of a detected asteroid on a collision course. There have been some successes in gaining attraction for this idea or establishing relevant international bodies, but also setbacks.

The establishment of the International Asteroid Warning Network (IAWN) and the Space Mission Planning Advisory Group (SMPAG) in 2014 had been a result of the roughly fifteen-years-long preceding efforts to transform proposals for better cooperation in planetary defense into semi-formalized bodies, currently called “groups”, under the United Nations Office for Outer Space Affairs (UNOOSA). The IAWN consists of astronomers observing and mapping asteroids and helps to translate their practices into formalized procedures ensuring smooth international cooperation. The SMPAG consists of nation-states representatives, usually from space agencies, and its objective is to draft options and *procedures* for any space-based mitigation response. These two groups were established following the three recommendations by the Association of Space Explorers that were adopted by Action Team 14 (an expert group to study Near Earth Object (NEO) mitigation options that were established following the UNISPACE III conference^5^ and then by the NEO Working Group in the UN Committee for Peaceful Use of Outer Space (COPUOS)^6^. In 2009, Action Team 14 submitted to the UN COPUOS Science and Technical Committee (STSC) a report with three critical recommendations in the policy section (para 23): “*(a) the need for shared NEO threat data, (b) the need for coordinated space mission planning, and (c) the need for an NEO oversight group representing the international community, which would definecriteria* *andpolicies* *to ensure a coordinated international response*”^7^.

While the first two recommendations were translated into the IAWN and the SMPAG respectively, the third recommendation was meant to be translated into the establishment of the Mission Authorization and Oversight Group (MAOG). However, nation-states decided that it would be premature to establish it at that time^8^. For now, the *criteria* to ensure a coordinated international response^7^ are currently under development in the SMPAG and are intended to serve as helpful procedures for coordinating states. However, proposing any *policies* remains a sensitive matter and is often viewed as outside the technical role of the SMPAG. On the other hand, the development of *criteria* and *procedures* is considered a less sensitive task as it seems to be technical without any political position. I would argue that developing *criteria* and *procedures* following the logic of regime development limits political decisions as well and, therefore, could finally become sensitive as well. One of the explanations for why MAOG was not established was the intention of the scientific community to authorize the decision about the planetary defense mission, or at least it could look like that, although the MAOG was considered as a middle decision-making body between the SMPAG and the UN Security Council^9^, by the Association of Space Explorers (ASE) report^10^, and finally also by Action Team 14 as well^7^. In fact, the proposals were explicit in stating that the MAOG “*must represent the international community as a whole”*^9^ and that the “*international community must speak with asingle voice, yet be responsive to reasonable questions and critiques from independent experts*”^10^. The sensitivity of the idea to provide the scientific community (that would speak on behalf of the international community and as a whole) with political legitimacy or somehow duplicate the role of the UN(SC) during this time was quite visible^11^. If *criteria* and *procedures* provide policymakers with a range of scenarios to choose from, they will fulfill SMPAG’s objectives while expanding policy options for political authorities. This approach maintains national sovereignty in decision-making but ensures scientific knowledge retains strong influence. This proposal mirrors negotiations between nations and scientists within the Intergovernmental Panel on Climate Change (IPCC). However, it must develop a clear, convergent normative argument of w*hat is the value we secure* outlining the ultimate objectives of the field to ensure mutual understanding.

The issue of mutual understanding regarding the necessity and responsibility to act was addressed as a procedural component of the regime. This was considered ‘procedural’ because, rather than establishing a norm, the emphasis was on creating a distinct group (MAOG) that would require decision-making procedures, potentially replacing UN procedures. Although norms are non-binding, they facilitate a shared comprehension of the problem, promoting a response to it. In contrast, adherence is integral to the design of procedures. Norms can be viewed as morally binding, preserving the sovereign maneuverability of political actors to implement their preferred policies. This approach is typically why states are more inclined to proceed with confidence-building measures rather than formal treaties.

Should the planetary defense community advocate for a “*single voice”* and if planetary defense requires “*coordinated international response”* as the scientific community collectively argues, the international community must reach a consensus on the following *normative* questions: *“What is the problem?”*, *“What is the appropriate behavior?”*, and *“What is the value we secure?”*.

## The way forward

The issue is not solely the risk of an asteroid impact, but also the emergence of new knowledge without consensus on its application. Mere procedural guidelines are insufficient; there needs to be generally internalized consensus to follow them and why. This new knowledge imposes a unique responsibility on sovereign nation-states, contributing to the re-articulation of the social contract. This aspect should not be employed to formulate sensitive policies within the planetary defense community. Instead, it should be used to establish norms that articulate why states need to address this issue, ensuring that discussions about policies are not considered sensitive.

The broader understanding of planetary defenseworthiness could create an additional bond among sovereign political entities. This bond would be predicated on existing scientific and technological partnerships related to planetary defense missions if political representatives deem it a matter of significance. Such significance would be due to its association with established national interests, exemplified by the cognitive resonance between planetary defense and national security policy, e.g., the need to cooperate on national security-sensitive technologies. However, perceptions of worthiness can also be forged through various other means such as business interests of the high-tech industry (supporting national industry), moral commitments to values (ratification of R2DE), a responsibility to act (reaffirmation of the commitment), direct national security concerns, and so on.

The USA, in its most recent NASA Planetary Defense Strategy and Action Plan, has chosen a different tone by mentioning in Goal 4.1 that *“NASA will leverage existing and new research to develop longterm principles for NASA’s planetary defense activities that maximize transparency, multilateral cooperation, and promote the benefits of applied planetary science for all humanity”*^12^. The necessity for multilateral cooperation in planetary defense, emphasizing transparency and collaboration for the benefit of humanity, is a narrative not present in the preceding 2018 strategy^13^ or in the 2016 strategy^14^ in such explicit wording. Moreover, the current USA strategy considers planetary defense *“a global challenge and a unique opportunity for engagement with the international community* [and] *an intrinsically global endeavor, drawing upon telescopes and capabilities on every continent and in orbit.”* This change in the narrative represents what is known in scholarly works as the *cosmopolitan responsible state*^15,16^ as it focuses on humanity as a benefactor and considers asteroid impact a global threat that depends on capabilities everywhere on the planet. In this narrative, planetary defense is conceived as an agenda for states imbued with an intrinsic sense of global responsibility—a value-laden concept. This marks the moment when a nation ceased to ignore the normative value of planetary defense, recognizing it simultaneously as a global issue and a national opportunity. This is a bid for responsible cosmopolitan governance in its clearest form. However, the USA aligns its interest in multilateral cooperation with the planetary defense agenda, epitomizing the cognitive components of a future security regime that is predicated on values essential to preserving humanity.

The Responsibility to Protect (R2P) doctrine, though not a formal regime but rather a political commitment endorsed by all UN member states does not specify procedures; instead, it posits that the highest value resides in human life, which must be protected. The doctrine articulates a moral responsibility of sovereign states and if they are unable to do so, it must be borne by the broader community^17^. One might argue that norm creation by expert groups is elitist. However, the proposed R2DE follows the same mechanism as R2P, ultimately leaving the decision to adhere to it with individual states, ensuring its political legitimacy. Moreover, R2P’s success lay in shifting foreign policy focus towards the sanctity of human life and its failure stems from the challenge to national sovereignty posed by the potential for military intervention in sovereign states. R2DE builds on the success of R2P and addresses its shortcomings by articulating the principles of a responsible foreign policy. This approach emphasizes action when a state possesses knowledge and capacity. It redefines traditional sovereignty, moving away from total political independence and towards a focus on autonomy–the capacity to act^18^.

The achievements of scientists and engineers embody a cosmopolitan responsibility that can be concretely translated into practical foreign policy^18^. The latest US strategy for planetary defense illustrates this point^12^; it recognizes a global challenge that must be met (the responsibility to act) and highlights the exceptional opportunity for international cooperation (capability building). This responsibility arises from newly acquired scientific knowledge, our technical ability to respond, and our political choice to act collectively as humanity. If the fundamental principle of the social contract between citizens and their sovereign is the latter’s capacity to ensure the safety of its citizens, then the planetary defense community holds the potential to lay the groundwork for a global social contract. Building on Wendt’s theories that the international system is shaped by the shared ideas and social interactions of states, rather than solely by material forces like military or economic power^19^, which have evolved into a significant branch of social constructivist political science. The nascent norm ‘Responsibility to Defend Earth’ has the substantial potential to exert a constitutive role, leading to policy changes even beyond the realm of planetary defense.

The epistemic community holds the potential to shape the normative component the value we secure–of the future security regime, and through interactions with states, infuse new substance into the very concept of national security and the rationale behind its necessity. It was once asserted that perceiving cosmopolitan security not merely as a normative preference but as a global categorical imperative is strategically essential for addressing inherently global challenges^20^.

We have a group of astronomers in IAWN to detect and define the threat *(what is the problem)* and a group of (mostly) engineers in SMPAG to propose a viable deflection mission. However, we are struggling to delineate a politically acceptable framework about the expected means of action (*what is the appropriate behavior)* within which we can define decision making procedures linked to possible mission designs. The current dynamics within SMPAG stem from a clear sensitivity toward engaging in discussions even related to “policy making,” which the group does not see as its mandate. Politically charged issues are often veiled behind the guise of “legal analysis.” The sensitivity is so significant that there is often no clear distinction perceived between “policy making” and “policy analysis” within the group. Policy options ought to be considered as critically and pragmatically as engineers’ mission designs.

Therefore, to break the deadlock, I argue that we do not need MAOG to decide and oversee the mechanics of the mission proposed by SMPAG, we need to deliberate on *the value*–to safeguard the planet as a whole–within the norm the Responsibility to Defend Earth. From there, we can extract elements for a future UN Security Council resolution on the action to defend the planet that could prompt the cognitive recognition by political authorities of planetary defense as an issue abundant with national opportunities. Policy ideas, or adopted policy options, by political authorities constituting appropriate behavior will become a natural cognitive component of an emerging regime.

## Building the norm responsibility to defend earth–R2DE

As norms typically arise from an informal consensus on the topic at hand, the following can be seen as an informal proposal for the foundations of the norm R2DE–‘Responsibility to Defend Earth.’ If the planetary defense community could agree on, adopt, and internalize these suggested values as its value system, they hold the potential to trigger cognitive reasoning within political authorities over its necessity. Consequently, these normative components could establish a foundation for a future binding instrument in international relations. But the question remains: *what is the value we secure?*

**Table:** 

**Pillar I**	*The responsibility to act*–scientists have produced knowledge that effectively rearticulates the social contract by revealing new insecurity and means to address it. The response can be authorized only by states with their sovereign authority. When a state, or a group of states, possesses the capability to respond, it has a moral (albeit non-binding) duty to do so, irrespective of whose territory might fall within the risk corridor, because the planetary defense is concerned with safeguarding the planet as a whole.
**Pillar II**	*The responsibility to develop the capability*–if a state, or a group of states, lacks the capability for the response, it has the responsibility to generate it and contribute whatever it can to bolster the collective planetary defense capabilities.
**Pillar III**	*The holistic understanding of planetary defense*–the eventual security regime should be designed as multigenerational, financially sustainable for decades, and beneficial to international scientific and industrial cooperation^21^.

Each pillar is essential for fulfilling the obligations imposed by the preceding one. Pillar III advocates for a paradigm shift, reconceptualizing planetary defense as a comprehensive endeavor that encompasses the scientific and industrial complex. It underscores the importance of long-term economic sustainability, which should not be compromised by an asteroid impact. Consequently, multilateral principles should guide cooperation in planetary defense in Pillars II and I. The principle of diffuse reciprocity is realized when countries are willing to deploy their capabilities in response to the threat (Pillar II). Where the responsibility to act in Pillar I is interpreted as the multilateral principle of indivisibility, it ensures all members of the multilateral security regime an equal guarantee of security, irrespective of their ability to contribute.

Addressing the aspirations articulated back in 2008^10^ that the “*international community must speak with a single voice, yet be responsive to reasonable questions and critiques from independent experts”* requires a consensus on the value we aim to safeguard. This is the agreement that politicians, scientists, experts, and the public need to establish: our knowledge dictates our responsibility to act. This fundamental accord, which should be jointly recognized by politicians, scientists, experts, and the public, underscores that our awareness necessitates action. As highlighted earlier, once there is agreement on the problem and a shared value system is embraced, the international community will see the appropriate response as both necessary and obligatory. Let the UN General Assembly vote over the norm in 2029, during the proposed International Year of Planetary Defence.
